# Membrane protein folding and quality control

**DOI:** 10.1016/j.sbi.2021.03.003

**Published:** 2021-08

**Authors:** Ben P. Phillips, Elizabeth A. Miller

**Affiliations:** MRC Laboratory of Molecular Biology, Francis Crick Avenue, Cambridge Biomedical Campus, Cambridge, CB2 0QH, UK

## Abstract

Membrane proteins account for a quarter of cellular proteins, and most are synthesised at the endoplasmic reticulum (ER). Insertion and folding of polypeptides in the membrane environment is prone to error, necessitating diverse quality control systems. Recent discoveries have demonstrated how forces act on the nascent chain during insertion, and revealed new translocon components and accessories that facilitate the correct biogenesis of substrates. Our understanding of one of the best studied quality control systems—ER-associated degradation—has been advanced through new structural and functional studies of the core Hrd1 complex, and through the discovery of a new branch of this degradative pathway. New data also reveal how cells resolve clogged translocons, which would otherwise be unable to function. Finally, new work elucidates how mitochondrial tail-anchored proteins that have been mistargeted to the ER are identified and destroyed. Overall, we describe an emerging picture of an increasingly complex quality control network.

## Introduction

For the bulk of the ∼25% of the human proteome that comprises integral membrane proteins, biogenesis begins at the endoplasmic reticulum (ER), where the core events of transmembrane domain (TMD) insertion, folding and quality control occur. Although some aspects of these processes are well understood, recent discoveries of new machineries that contribute to these events has shed new light on an increasingly complex and adaptable system that drives high-fidelity membrane protein biogenesis. In this review, we will focus on new findings concerning integration of TMDs into the bilayer, the recognition and extraction of aberrant membrane proteins, and the folding of lumenal domains at the ER.

## Membrane targeting and TMD insertion

**Figure 1:**
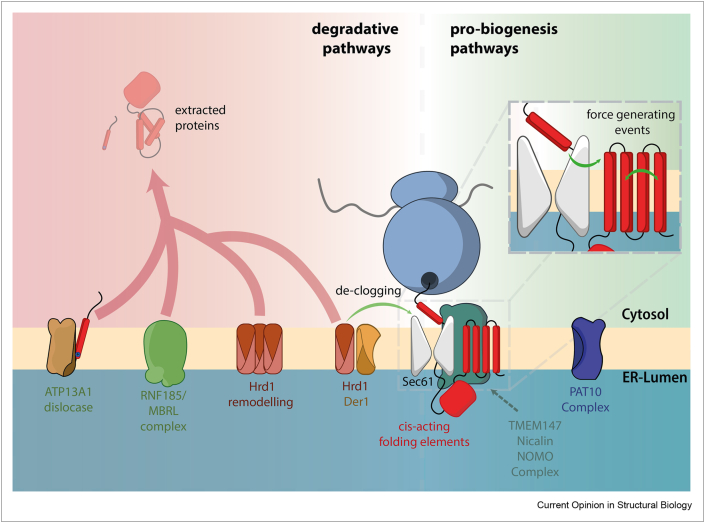
**Cellular machineries that drive quality control at the ER**. Recent work has revealed new pathways in degradative quality control pathways, and in the pro-biogenesis machinery required for substrate insertion and folding. Degradative processes include the ATP13A1 dislocase that removes mislocalised mitochondrial substrates from the ER; the RNF185/MBRL complex that drives ERAD of integral membrane proteins; the ERAD component Hrd1, for which a new structure reveals a membrane thinning channel produced by two V-shaped cavities in Hrd1/Der1, and the capacity for Hrd1 to resolve clogging of the Sec61 translocon. New pro-biogenesis events include force generating events that contribute to polytopic membrane protein insertion; cis-acting elements that contribute to lumenal domain folding; the TMEM147/Nicalin/NOMO translocon associated complex and the PAT10 complex, both of which promote polytopic membrane protein biogenesis.

Membrane proteins are initially delivered to the ER when translating ribosomes are recognised by the signal recognition particle via a hydrophobic nascent chain. This ternary complex docks at the ER membrane via interactions with the signal recognition particle receptor, and the ribosome is handed off to an hourglass shaped complex called the Sec61 translocon. Sec61 contains a central pore that traverses the membrane as well as a lateral gate that can open to provide access to the lipid bilayer. As the emerging nascent polypeptide enters the Sec61 translocon, it samples the lipid environment, and stretches of high hydrophobicity are thought to partition through the lateral gate to embed in the membrane (Figure 1a).

The core process of TMD insertion through the Sec61 lateral gate has been thoroughly dissected by a combination of genetics, biochemistry and structural biology [8]. However, the biophysical basis of interactions between the nascent chain, the translocon and the lipid bilayer that drive insertion is only now coming to light. Recent work has harnessed the complementary techniques of coarse-grain molecular dynamics and ‘force profiling’ to reveal with single amino acid resolution how the nascent chain generates force to aid TMD insertion [22]. This discovery built on the observation that integration of TMDs into the membrane and their subsequent folding generates a force that can be measured using arrest peptides [7,9,23]. The new work revealed that specific interactions between multiple TMDs in the bilayer and sequence elements flanking TMDs can also make significant contributions to force generation during insertion at the ER. The forces generated during TMD insertion may contribute a pulling force on the nascent chain that could overcome transient stalls associated with specific amino acid sequences and folding bottlenecks. Such events are likely to be especially important during biogenesis of multipass membrane proteins, and may contribute to co-translational quality control [14] that might be influenced by tension on the nascent chain during TMD insertion [26].

## Folding of multipass membrane proteins

After the sequential insertion of individual TMDs into the bilayer, the helices of multipass membrane proteins commonly assemble to form a helical bundle that corresponds to the ‘folded’ structure. Recent investigations using single-molecule magnetic tweezer approaches have complimented force profile measurements to reveal the dynamics of helical hairpin formation during helical bundle formation for the first time [6]. Achieving this final state is often nontrivial as many TMDs contain charged or polar residues that are required for function but that destabilise the protein in the membrane environment. As a result, many individual TMDs are unstable or poorly inserted into the membrane in the absence of their neighbours [10]. Recent structural characterisation of an expanded translocon apparatus comprising Sec61, TMCO1, CCDC47 and the Nicalin-TMEM147-NOMO complex suggests that an array of accessories around the Sec61 translocon may help to shelter or insert TMDs of multipass membrane proteins [16]. In parallel, recent structures of the TMCO1-related ER-membrane complex have resulted in proposals for the role of the complex in the insertion and potential chaperoning of TMD substrates [2,19,24,27]. In addition, a novel protein complex called the PAT10 complex, comprising PAT10 and Asterix, has been demonstrated to engage poorly hydrophobic TMDs to promote the biogenesis of multiple multipass membrane protein substrates [5]. The complex binds to unshielded hydrophilic or polar residues before releasing the substrate once all the TMDs have entered the bilayer and folding can be completed. In sum, these recent discoveries show that previous models of the co-translational biogenesis process have not captured the complex array of accessory factors required for high efficiency and fidelity in inserting substrates *in vivo*.

In addition to helical bundle formation, lumenal and cytosolic domains must also fold to form the final active protein, although our understanding of the interplay between these different folding events remains sparse. Even theoretically simple questions about the timings and order of events during lumenal domain folding have only recently been answered. *Kadokura et al* have revealed how the primary sequence of the nascent chain can influence the folding of soluble domains at the ER in surprising ways [12]. In studying the folding of the low-density lipoprotein receptor by investigating the timing of disulphide bond formation in the large lumenal domain, the authors found an intricate and precisely controlled process. Initial disulphides form as the domain is inserted into the lumen, and isomerisation is rapidly accelerated upon synthesis of upstream beta propellers which are known to fold rapidly. The isomerisation process is concurrent with the folding of the beta propellers and ensures that the correct bonds have all been formed before the lumenal domain becomes glycosylated. This study suggests that post-translational modifications in the ER might be controlled by *cis* actors at a primary sequence level, namely rapidly folding domains. This process could represent an additional level of regulation during the nonvectorial conversion of a primary amino acid sequence into a complex 3D structure.

## Destruction of aberrant membrane proteins

Given the complex folding requirements of multipass membrane proteins and the requisite temporal coordination, it's no surprise that multiple steps are prone to error. Accordingly, a range of quality control pathways supervise the process by identifying and degrading aberrant substrates. In eukaryotes, the central driver of ER quality control is the well-characterised proteasome-mediated ER-associated degradation (ERAD) pathway [11,18]. Recent discoveries that we highlight here reveal the complex and interconnected nature of membrane protein insertion and quality control at the ER, where cells must balance the timing of protein biogenesis and productive folding with rescue when things go wrong. For example, when TMD insertion into the bilayer is not energetically favourable or the translocon complex becomes otherwise occluded, the channel must be ‘de-clogged’ before it is available for a new round of insertion. This is a general principle that applies across organelles, and several ‘de-clogging’ agents act at different organelles [13,31]. Previous work in yeast identified the protease, Ste24, as the de-clogger of the Sec61 translocon [1]. New evidence indicates that Ste24 and a central component of yeast ERAD, Hrd1 [28], act redundantly to promote degradation of aberrant proteins that stably associate with the translocon. Hrd1 can also compensate for loss of Dfm1, another core ERAD component that is required for the extraction of membrane-embedded substrates [20,21]. In this latter case, the requirement for Hrd1 is revealed by a cellular adaptation process that involves overexpression of Hrd1 and the selective degradation of its otherwise obligate binding partner Hrd3 [21]. In accordance with previous evidence that Hrd1 overexpression can bypass deletion of upstream genes involved in retrotranslocation of misfolded lumenal substrates [3], it appears that Hrd1 can act as a multipurpose pressure valve to enable extraction of a wide variety of substrates from the ER.

Hrd1 is an integral membrane protein, which contains a V-shaped channel that penetrates into the membrane from the cytosol [29]. An updated structural study of Hrd1 in complex with Hrd3, Der1, Usa1 and Yos9 reveals physiologically relevant structures [34]. The channel is formed by a combination of Hrd1 and Der1 which each contributes one V-shaped cavity originating in the cytosol and the ER lumen, respectively. Together, the two cavities appear to thin the membrane, reducing the energetic penalty for substrate translocation. The twin V-shaped cavities are reminiscent of structures observed in the TMCO1-containing extended translocon [16], suggesting that partial membrane thinning may play a role in both the insertion and extraction of substrates at the ER.

Recent cryo-EM structures have also shed light on a parallel form of quality control involving the extraction of mistargeted membrane proteins [17]. After the identification of ATP13A1 as factor required for the efficient extraction of mislocalised mitochondrial proteins at the ER, *McKenna* *et* *al.* demonstrated that the ATPase is a *bona fide* dislocase through biochemical reconstitution experiments. The authors turned to cryo-EM to understand how the complex was able to select specific TMDs and extract them from the membrane, considering that most other members of this protein family transport ions and phospholipids. The complex was trapped in multiple different states, revealing different stages in the extraction process, including the visualisation of a bound TMD. The resulting structures indicated that, unlike other members of the P5-type ATPases, the binding site was unable to coordinate cations. Instead, a negatively charged binding cleft contributes to interaction with a cluster of positive charges proximal to the transmembrane domain on substrates on the lumenal side. A conformational change then exposes the binding cleft to the membrane and the cytosol simultaneously, permitting extraction of the TMD from the ER membrane. ATP13A1 is now part of a growing list of characterised dislocases involved in quality control at the membrane including Msp1 [4,15,25,33] and Hrd1 [30].

Finally, even among the well-characterised core process of ERAD, new discoveries are still being made that deepen our understanding of the complex and adaptable mammalian ERAD network. How aberrant substrates are recognised within the lipid bilayer and extracted into the cytosol remains poorly understood. New work from *van de Weijer* *et* *al* investigated the degradation of two superficially similar reporters that both contained a single TMD and an ER lumenal amphipathic α-helix [32]. Remarkably, the substrates (derived from yeast and human proteins) were degraded by different ERAD branches: the yeast-derived substrate was degraded by the canonical TEB4 ligase complex, whereas the mammalian substrate used a new ERAD complex comprising RNF185, TMUB1 and TMUB2 and Membralin (MBRL). Experiments using chimeras revealed that the specificity determinants were encoded in the TMD for the TEB4 pathway, whereas the RNF185/MBRL complex recognised a more complex array of cues. The mechanistic basis for substrate recognition in ER quality control is one of the key questions that remains poorly understood.

## Perspectives

Recent work has revealed increasing levels of complexity and sensitivity in regulation and quality control at the ER. Although full mapping of the energy landscape of membrane protein folding is still beyond our reach, we have new insight into diverse cellular machineries that facilitate this process. Moving beyond the parts list of which proteins act in specific processes during membrane protein biogenesis, new assays like force profiling and single-molecule refolding experiments will continue to provide fine-grained views of the folding pathways of different substrates. One of the key questions moving forward is how the timing of these different, often competing, pathways are coordinated to maximise productive folding and still protect the cell from the consequences of misfolding. Understanding the molecular hand-off between sequential machineries will be an important area for future investigation.

## Funding

This work was supported by the UK 10.13039/501100000265Medical Research Council (MRC_UP_1201/10 to EAM).

## Conflict of interest statement

Nothing declared.
